# Dynamic Arterial Elastance in Predicting Arterial Pressure Increase After Fluid Challenge During Robot-Assisted Laparoscopic Prostatectomy: A Prospective Observational Study

**DOI:** 10.1097/MD.0000000000001794

**Published:** 2015-10-16

**Authors:** Hyungseok Seo, Yu-Gyeong Kong, Seok-Joon Jin, Ji-Hyun Chin, Hee-Yeong Kim, Yoon-Kyung Lee, Jai-Hyun Hwang, Young-Kug Kim

**Affiliations:** From the Department of Anesthesiology and Pain Medicine, Seoul National University Hospital, Seoul, Republic of Korea (HS); Department of Anesthesiology and Pain Medicine, Asan Medical Center, University of Ulsan College of Medicine, Seoul, Republic of Korea (Y-GK, S-JJ, J-HC, J-HH, Y-KK); and Department of Anesthesiology and Pain Medicine, Hangang Sacred Heart Hospital, Hallym University College of Medicine, Seoul, Republic of Korea (H-YK, Y-KL).

## Abstract

During robot-assisted laparoscopic prostatectomy, specific physiological conditions such as carbon dioxide insufflation and the steep Trendelenburg position can alter the cardiac workload and cerebral hemodynamics. Inadequate arterial blood pressure is associated with hypoperfusion, organ damage, and poor outcomes. Dynamic arterial elastance (Ea) has been proposed to be a useful index of fluid management in hypotensive patients. We therefore evaluated whether dynamic Ea can predict a mean arterial pressure (MAP) increase ≥ 15% after fluid challenge during pneumoperitoneum and the steep Trendelenburg position.

We enrolled 39 patients receiving robot-assisted laparoscopic prostatectomy. Fluid challenge was performed with 500 mL colloids in the presence of preload-dependent conditions and arterial hypotension. Patients were classified as arterial pressure responders or arterial pressure nonresponders according to whether they showed an MAP increase ≥15% after fluid challenge. Dynamic Ea was defined as the ratio between the pulse pressure variation and stroke volume variation. Receiver operating characteristic curve analysis was performed to assess the arterial pressure responsiveness after fluid challenge during robot-assisted laparoscopic prostatectomy.

Of the 39 patients, 17 were arterial pressure responders and 22 were arterial pressure nonresponders. The mean dynamic Ea before fluid challenge was significantly higher in arterial pressure responders than in arterial pressure nonresponders (0.79 vs 0.61, *P* < 0.001). In receiver operating characteristic curve analysis, dynamic Ea showed an area under the curve of 0.810. The optimal cut-off value of dynamic Ea for predicting an MAP increase of ≥ 15% after fluid challenge was 0.74.

Dynamic Ea can predict an MAP increase ≥ 15% after fluid challenge during robot-assisted laparoscopic prostatectomy. This result suggests that evaluation of arterial pressure responsiveness using dynamic Ea helps to maintain an adequate arterial blood pressure and to improve perioperative outcomes in preload-dependent patients receiving robot-assisted laparoscopic prostatectomy under pneumoperitoneum and in the steep Trendelenburg position.

## INTRODUCTION

Robot-assisted laparoscopic prostatectomy is widely performed due to its many advantages, including a reduced need for blood transfusion and fewer surgical complications compared with conventional open prostatectomy.^1^ However, a specific physiological condition is induced by the carbon dioxide insufflation required and the steep Trendelenburg position used during robotic prostatectomy. Previous reports have shown that pneumoperitoneum and the Trendelenburg position can affect the cardiac workload^2,3^ and cerebral hemodynamics.^4,5^ Additionally, the upper age limit of patients receiving robotic prostatectomy is increasing,^6^ and patients with prostate cancer have increased cardiovascular risks.^7^ Therefore, further consideration of hemodynamic management, including fluid therapy, is needed during the perioperative period for patients undergoing robotic prostatectomy.

Maintenance of adequate arterial blood pressure is essential to avoid tissue hypoperfusion, organ damage, and subsequent poor outcomes in noncardiac surgery.^8–10^ When the mean arterial pressure (MAP) decreases in preload-dependent conditions, fluid administration is generally regarded as an initial therapy. Interestingly, dynamic arterial elastance (Ea), which is defined as the ratio between the pulse pressure variation (PPV) and stroke volume variation (SVV), has been proposed to be a useful index of fluid management in hypotensive patients.^11,12^ However, there are no reports on the ability of dynamic Ea to predict arterial pressure responsiveness under the special surgical conditions generated by the steep Trendelenburg position and carbon dioxide pneumoperitoneum.

In our present study, we hypothesized that dynamic Ea could be a useful predictor of arterial pressure responsiveness in robot-assisted laparoscopic prostatectomy. To test our hypothesis, we evaluated whether dynamic Ea could predict an MAP increase ≥ 15%^13^ in preload-dependent conditions (SVV > 10%)^14^ and with arterial hypotension (MAP < 65 mm Hg or systolic arterial pressure <90 mm Hg)^8^ in patients receiving robot-assisted laparoscopic prostatectomy under carbon dioxide pneumoperitoneum and in the steep Trendelenburg position.

## METHODS

### Patients

This prospective observational clinical study was conducted between June 2014 and September 2014. The study protocol was approved by the Asan Medical Center Institutional Review Board (approval number: 2014–0522) and registered on an international clinical trials registry platform (http://cris.nih.go.kr, KCT0001131). A total of 42 male patients who were scheduled for robot-assisted laparoscopic prostatectomy due to prostate cancer were enrolled and written informed consent was obtained from all patients. The exclusion criteria were as follows: < 20 or > 80 years of age; medical history of arrhythmia, valvular heart disease, ischemic heart disease, or left ventricular ejection fraction < 40%; any pulmonary disease or abnormal preoperative chest radiography; renal disease (serum creatinine > 1.4 mg/dL or receiving dialysis); new-onset arrhythmia after anesthesia induction; failure of arterial catheterization; or an unexpected severe intraoperative hemodynamic change.

### PPV

The PPV was defined as 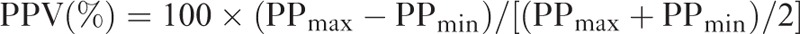


where PP_max_ and PP_min_ indicate the maximal and minimal pulse pressure in a respiratory cycle, respectively. Data on arterial blood pressure were transferred to the Phillips IntelliVue MP90 monitoring system (Philips, Best, The Netherlands). The PPV was automatically calculated and averaged >4 consecutive cycles of 8 s.

### SVV

A specialized hemodynamic monitoring system (EV1000 clinical platform, Edward Lifesciences Corp, Irwin, CA) was connected to the arterial line via FloTrac™ sensors (Edwards Lifesciences Corp). Cardiac output and stroke volume were calculated by real-time arterial waveform analysis. The SVV was computed every 20 s according to the following formula: 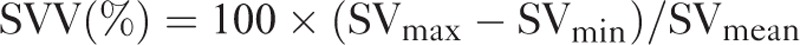


After zeroing to atmosphere, cardiac output, stroke volume, and SVV values were obtained continually by arterial waveform analysis. SV_max_, SV_min_, and SV_mean_ indicate the maximal, minimal, and mean stoke volume in a respiratory cycle, respectively.

#### Dynamic Ea

Dynamic Ea was defined as the PPV/SVV ratio and was calculated from the average of 3 consecutive measurements of the SVV and PPV at a specific time point. In a previous report,^12^ the PPV measured by the IntelliVue MP90 and the SVV measured by the EV1000 were used to evaluate fluid responsiveness in robotic prostatectomy.

### Study Protocol

General anesthesia and patient monitoring were performed according to our institutional standards. Intraoperative monitoring included electrocardiography, intra-arterial blood pressure, end-tidal carbon dioxide concentration, and peripheral oxygen saturation. Anesthesia was induced with thiopental sodium 5 mg/kg and target-controlled infusion of remifentanil (Orchestra^®^ Base Primea; Fresenius Kabi, Bad Homburg, Germany) with an effect site concentration of 3 ng/mL. Rocuronium bromide 0.6 mg/kg was given to facilitate tracheal intubation. Anesthesia was maintained with 1vol% to 2vol% sevoflurane and 50% oxygen in medical air. The effect site concentration of remifentanil target-controlled infusion was adjusted to between 3 and 8 ng/mL. The depth of anesthesia was monitored using the bispectral index (A-1050 Monitor; Aspect Medical Systems, Newton, MA), which was maintained between 40 and 60. Sevoflurane administration and remifentanil target-controlled infusion were intermittently adjusted during surgery according to the bispectral index or hemodynamic parameters but were not changed during fluid challenge or hemodynamic measurements. Mechanical ventilation using an anesthetic machine (Primus^®^; Dräger, Lübeck, Germany) was performed using a fixed tidal volume of 8 mL/kg (ideal body weight) and a respiratory rate of 10 to 16/min to maintain an end-tidal carbon dioxide concentration of between 35 and 40 mm Hg. A positive end-expiratory pressure of 8 cm H_2_O was applied equally in all patients. During surgery, all patients were placed in the Trendelenburg position at 35° and pneumoperitoneum was achieved by continuous carbon dioxide insufflation maintaining an intra-abdominal pressure of 12 cm H_2_O.

After the induction of anesthesia, intra-arterial pressure was continuously monitored using radial arterial catheterization, and an additional intravenous route for fluid challenge was secured with a 16-gauge catheter. After zeroing to atmosphere, an indwelling radial artery catheter was simultaneously connected to the Philips IntelliVue MP90 monitoring system and the EV1000. Intraoperative basal fluid was restricted with continuous infusion of 2 mL/kg/h of crystalloid solution. Hemodynamic parameters were measured at 4 time points: 10 min after the Trendelenburg position was adopted and carbon dioxide insufflation (T1), just before fluid challenge (T2), 3 min after fluid challenge (T3), and at the time of skin closure (T4). T1 was regarded as the baseline for the hemodynamic parameters. T2 was determined by the presence of the preload-dependent condition, which is defined as the maintenance of SVV > 10% for >10 min,^14^ and arterial hypotension (MAP < 65 mm Hg or systolic arterial pressure < 90 mm Hg).^8^

Fluid challenge was carried out with 500 mL 6% hydroxyethyl starch (Volulyte^®^; Fresenius Kabi, Bad Homburg, Germany) >20 min via an infusion pump. During fluid challenge (between T2 and T3), the respiratory rate or anesthetic concentration was not altered. When MAP was not maintained at 65 mm Hg or higher despite fluid challenge, 5 to 10 mg intravenous ephedrine was administered after evaluating the dynamic Ea.

Postoperative pulmonary complications, defined as the presence of abnormal findings on chest radiography (atelectasis, pulmonary infiltration, pulmonary vascular congestion, pulmonary edema, and pleural effusion), and abnormal findings in laboratory tests were evaluated until postoperative day 7.

### Outcome Measurement

Patients were classified as arterial pressure responders or arterial pressure nonresponders according to whether they achieved an MAP increase ≥ 15% after fluid challenge.^13^ At each time point, the cardiac index, MAP, heart rate, stroke volume index, PPV, and SVV were measured. All variables are recorded directly from the hemodynamic monitoring system, except for dynamic Ea, which was derived and calculated from the PPV and SVV.

### Statistical Analysis

Sample size was determined using the difference between the area under the curve of 0.75 (alternative hypothesis that dynamic Ea can predict arterial pressure responsiveness after fluid challenge) and 0.5 (null hypothesis). Assuming a type I error of 0.05 and a desired power of 0.80, 38 patients were required for the analysis. Expecting a dropout rate of 10%, 42 patients were enrolled. Normality of data was tested using the Shapiro–Wilk test. Differences in patient characteristics, preoperative laboratory values, and intraoperative and postoperative data between arterial pressure responders and arterial pressure nonresponders were compared using an unpaired *t* test or the Mann–Whitney rank sum test. Categorical data between the 2 groups were compared using the chi-square test or Fisher's exact test. To simultaneously compare the change in hemodynamic variables after fluid challenge and the difference between arterial responders and arterial nonresponders, 2-way repeated measure analysis of variance was used. Receiver operating characteristic curve analysis was performed to assess the arterial pressure responsiveness after fluid challenge for each hemodynamic variable (dynamic Ea, PPV, SVV, MAP, stroke volume index, and heart rate at T2). The optimal cut-off value was determined using a value based on the Youden index, which was calculated as a maximum (sensitivity + specificity – 1). An area under the curve of > 0.75 was considered to show good prediction.^15^ All results are expressed as mean ± SD or number (proportion). *P* < 0.05 was considered statistically significant. Statistical analysis was performed using MedCalc^®^ version 13.2.0 (MedCalc Software, Ostend, Belgium) or SigmaPlot 10.0 (Systat Software, Inc, San Jose, CA).

## RESULTS

**FIGURE 1:**
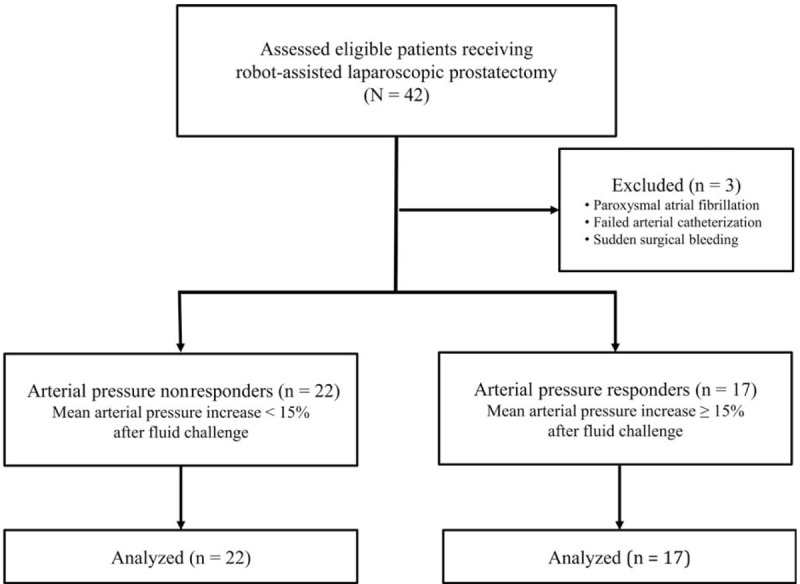
Study flow chart. Arterial pressure nonresponders were defined by a mean arterial pressure increase of < 15% after fluid challenge, whereas arterial pressure responders were defined by a mean arterial pressure increase of ≥ 15% after fluid challenge in patients undergoing robot-assisted laparoscopic prostatectomy.

A total of 42 patients were initially eligible for this study. However, 3 patients were excluded: 1 developed paroxysmal atrial fibrillation during surgery, another had arterial catheterization failure, and the remaining patient had sudden surgical bleeding and a subsequent severe intraoperative hemodynamic change. Finally, 39 patients were enrolled (Fig. 1). The demographics and perioperative data of arterial pressure nonresponders and arterial pressure responders are summarized in Table 1. There were no significant differences in variables between arterial pressure nonresponders and arterial pressure responders in preload-dependent patients receiving robotic prostatectomy under carbon dioxide pneumoperitoneum and in the steep Trendelenburg position.

**TABLE 1:**
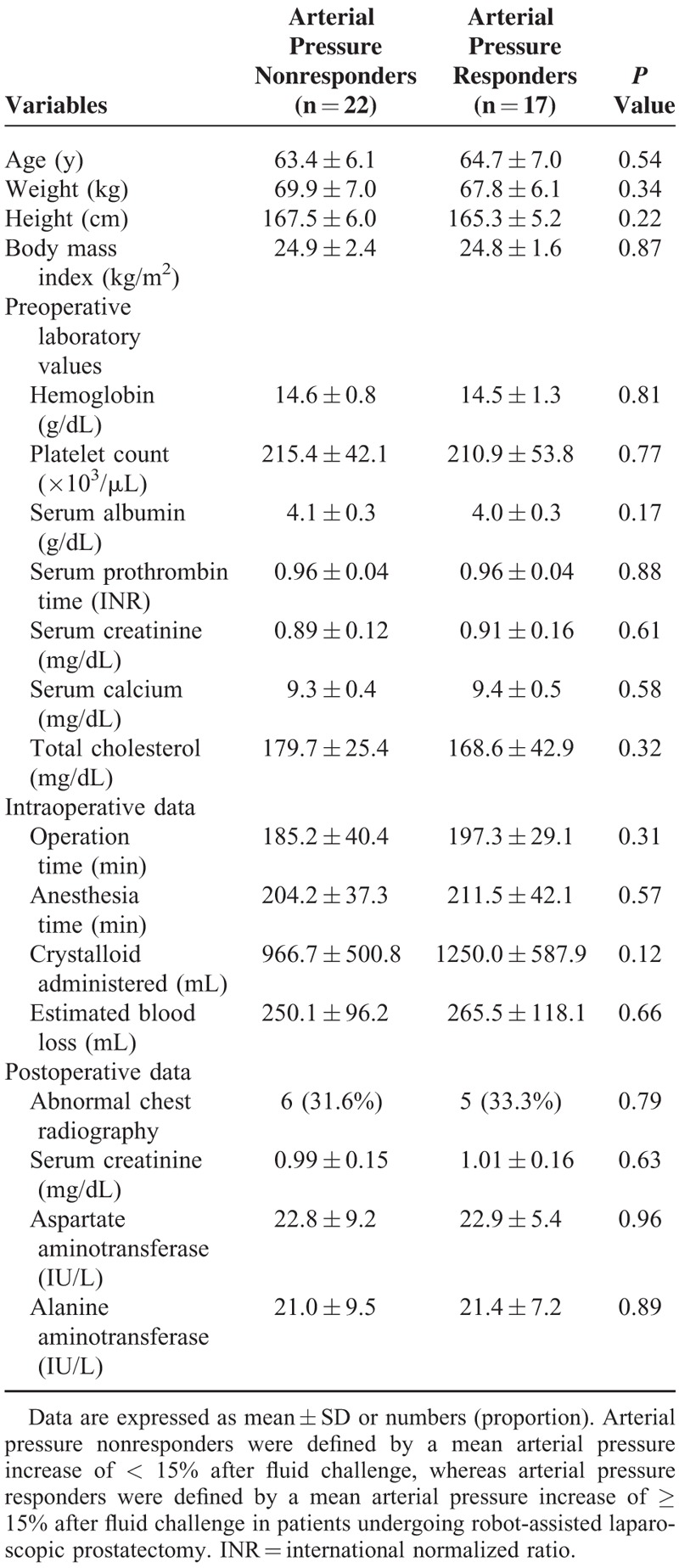
Demographic and Perioperative Data Between Arterial Pressure Nonresponders and Responders

**FIGURE 2:**
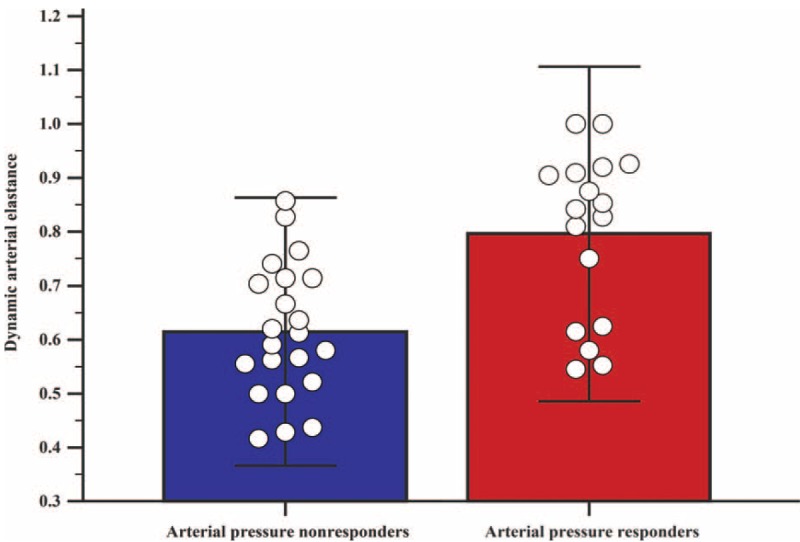
Comparison of dynamic arterial elastance between arterial pressure nonresponders (blue box) and arterial pressure responders (red box) in patients undergoing robot-assisted laparoscopic prostatectomy. Arterial pressure nonresponders were defined by a mean arterial pressure increase of < 15% after fluid challenge, whereas arterial pressure responders were defined by a mean arterial pressure increase of ≥ 15% after fluid challenge. Individual data are expressed as blank circles. The mean dynamic arterial elastance of each group (upper border of each box) was significantly different between nonresponders and responders (*P* < 0.001).

Hemodynamic parameters before and after fluid challenge are listed in Table 2. Larger increases in the MAP, stroke volume index, and cardiac index after fluid challenge were seen in arterial pressure responders than in arterial pressure nonresponders. A statistically significant difference in the mean dynamic Ea before fluid challenge (T2) was seen between arterial pressure nonresponders and arterial pressure responders (0.61 vs 0.79, *P* < 0.001; Fig. 2).

**TABLE 2:**
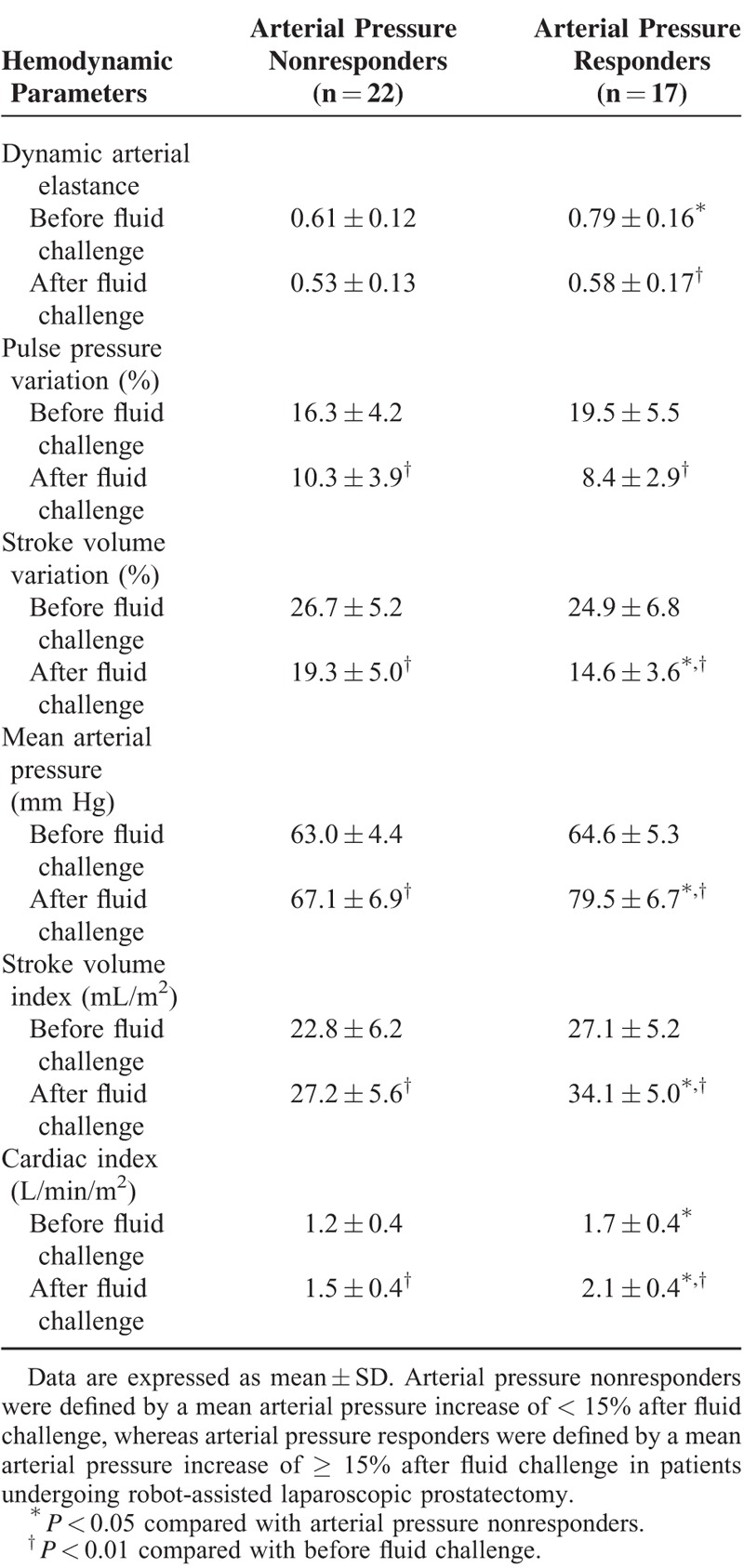
Changes in Hemodynamic Parameters After Fluid Challenge Between Arterial Pressure Nonresponders and Responders

The area under the curve of each hemodynamic variable for the prediction of arterial pressure responsiveness after fluid challenge is provided in Table 3. The area under the curve of dynamic Ea for predicting arterial pressure responders was 0.810 (95% confidence interval = 0.650–0.916, *P* < 0.001). Various cut-off values are listed in Table 4, and the optimal cut-off value of dynamic Ea for predicting an MAP increase of ≥ 15% after fluid challenge was 0.74 in patients receiving robotic prostatectomy under carbon dioxide pneumoperitoneum and in the steep Trendelenburg position.

**TABLE 3:**
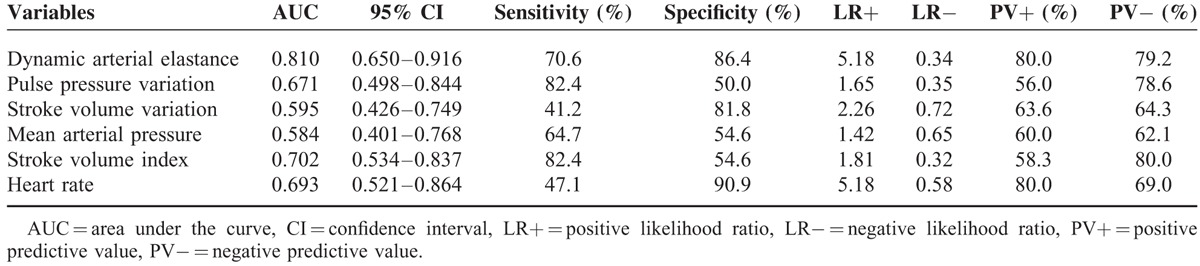
Receiver Operating Characteristic Curve Analysis of the Hemodynamic Variables Predicting Arterial Pressure Responsiveness After Fluid Challenge

**TABLE 4:**
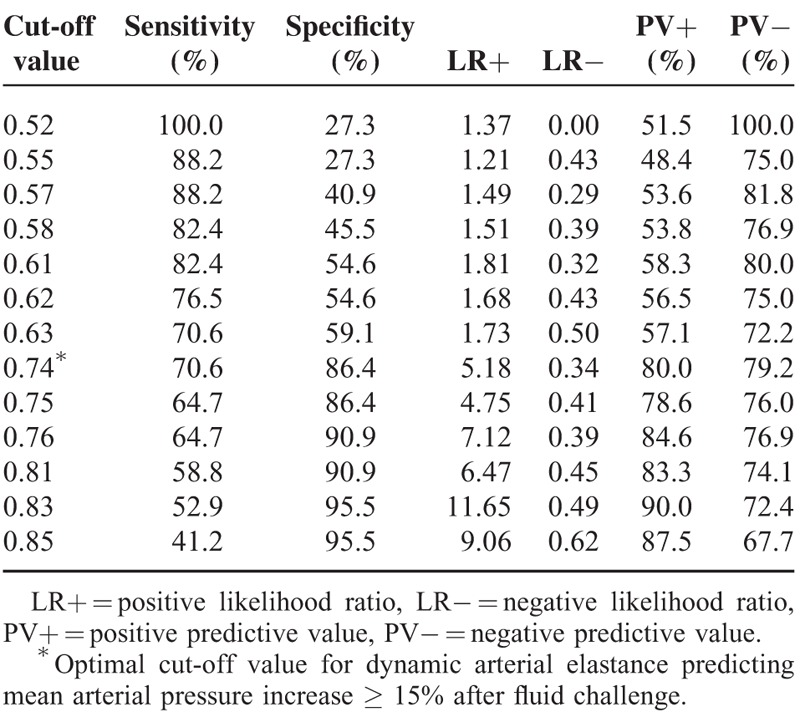
Cut-off Values for the Dynamic Arterial Elastance Predicting Arterial Pressure Responsiveness After Fluid Challenge

## DISCUSSION

In our present study, we have shown that dynamic Ea can predict an increase in the MAP of ≥ 15% after fluid challenge in preload-dependent patients receiving robot-assisted laparoscopic prostatectomy under carbon dioxide pneumoperitoneum and in the steep Trendelenburg position. In addition, we found that a dynamic Ea value of > 0.74 was associated with an MAP increase of ≥ 15% during robot-assisted laparoscopic prostatectomy.

The steep Trendelenburg position and pneumoperitoneum increase right- and left-side cardiac filling pressure.^2,16^ Significant changes in the MAP, stroke volume, and systemic vascular resistance are associated with carbon dioxide insufflation and/or the head-down position.^16^ These earlier findings suggest that special surgical conditions during robotic prostatectomy can increase cardiac workload. In addition, cerebral perfusion pressure can be affected by increased intracranial pressure and intrathoracic pressure in patients receiving robotic prostatectomy under carbon dioxide pneumoperitoneum and in the steep Trendelenburg position.^17^ Furthermore, in elderly patients undergoing robotic prostatectomy, cardiovascular function can be compromised.^2,16,18^ Importantly, inadequate arterial blood pressure is associated with tissue hypoperfusion, organ damage, and poor outcomes.^8–10^ Thus, adequate maintenance of intraoperative arterial blood pressure is important in patients receiving robotic prostatectomy under carbon dioxide pneumoperitoneum and in the steep Trendelenburg position.

The SVV and PPV are widely used as fluid responsiveness indexes.^19,20^ During positive pressure ventilation, cyclic changes in intrathoracic pressure can induce changes in stroke volume and arterial pulse pressure, and such variation can consequently be used as a dynamic volume index. However, clinical situations causing intrathoracic pressure changes can induce variations in right atrial pressure, thereby changing intrathoracic blood volume during positive pressure ventilation.^21^ In robot-assisted laparoscopic prostatectomy, for a good surgical field, the patient must be placed in a steep Trendelenburg position (25°–45° head down).^21^ In this specific position, the abdominal organs are pulled away from the operative field by gravity, causing an intrathoracic pressure increase. Moreover, additional carbon dioxide insufflation may affect venous return to the right atrium and exaggerate intrathoracic pressure changes. Therefore, fluid responsiveness assessed by the SVV or PPV may be influenced by the increased intrathoracic pressure and be less accurate during robotic prostatectomy.^22,23^ In addition, to evaluate the stroke volume index after a fluid challenge (ie, volume responsiveness), a relatively constant arterial vascular tone would be required.^24,25^ Specific surgical conditions such as carbon dioxide insufflation and the steep Trendelenburg position can decrease venous return and predispose the patient to a decreased cardiac afterload,^26^ possibly reducing the predictability of the fluid responsiveness of both the SVV and PPV. Based on these considerations, the predictability of the arterial pressure responsiveness of the SVV and PPV can also be influenced, at least in part, by the increased intrathoracic pressure and decreased cardiac afterload during robotic prostatectomy.

However, dynamic Ea, which is not an absolute value but a ratio between the PPV and SVV, is a useful index of fluid management in hypotensive patients under spontaneous breathing.^27^ In addition, dynamic Ea reflects the arterial vascular tone.^19,28^ Taken together, dynamic Ea is thought to be minimally influenced by changes in intrathoracic pressure and cardiac afterload. In previous studies, dynamic Ea was used to detect the need for vasopressor administration in preload-dependent patients.^20,29^ In our present study, we found that dynamic Ea can predict an increase in the MAP of ≥ 15% after a fluid challenge in preload-dependent patients receiving robotic prostatectomy under carbon dioxide pneumoperitoneum and in the steep Trendelenburg position. We considered that evaluation of the arterial pressure responsiveness using dynamic Ea could help to maintain adequate arterial blood pressure and to improve perioperative outcomes in patients receiving robotic prostatectomy.

We obtained various cut-off values from 0.52 to 0.85 and found that the optimal cut-off value of dynamic Ea to predict an MAP increase of ≥ 15% after fluid challenge was 0.74. The optimal cut-off value of 0.74 can be used clinically as a good guide for predicting arterial pressure responsiveness after volume loading during robotic prostatectomy. The optimal cut-off value of dynamic Ea for predicting an MAP increase after fluid challenge has been reported to be 0.9 to 1.25 in several clinical situations.^13,27^ The dynamic Ea cut-off value during robotic prostatectomy in our current analyses was measured as 0.74, which was lower than that of previous reports.^13,27^ Although it would be difficult to compare our results with those of previous studies, the SVV is thought to be more affected by intrathoracic pressure changes than the PPV in patients under carbon dioxide pneumoperitoneum and in the steep Trendelenburg position.^22^ Increased intrathoracic pressure could consequently increase both the SVV and PPV.^23^ However, the SVV is reported to be more influenced by increased intra-abdominal pressure than the PPV.^22^

There were several possible limitations to our present study. First, we evaluated fluid responsiveness using the MAP increase. Although cardiac output can be more important in preventing tissue hypoperfusion than MAP, measurement of cardiac output using pulmonary arterial catheterization or transesophageal echocardiography is clinically invasive and difficult in routine daily practice. The insertion of a pulmonary arterial catheter is associated with fatal complications.^30^ Also, use of intraoperative transesophageal echocardiography requires a trained physician and is expensive and invasive.^31,32^ Compared with cardiac output measurement using pulmonary arterial catheterization or transesophageal echocardiography, dynamic Ea can be measured more simply and less invasively. Second, SVV and PPV values were obtained at different times by their respective measurement devices. The SVV was calculated from the FloTrac^®^ sensor/EV1000 device every 20 s, whereas the PPV was obtained from the Philips MP90 monitor every 8 s. However, to decrease the time difference, we recorded all SVV or PPV values for 3 consecutive measurements at specific time points and used an average value. Therefore, we consider that the effect of the time difference on dynamic Ea is minimal.

In conclusion, the dynamic Ea, which is simply calculated from the SVV and PPV, can be predictive of arterial pressure responsiveness to fluid challenge in robot-assisted laparoscopic prostatectomy. The optimal cut-off value of dynamic Ea for predicting an MAP increase of ≥ 15% after fluid challenge is 0.74. Our present findings provide useful information for future fluid management protocols in patients with preload-dependent condition and arterial hypotension during carbon dioxide pneumoperitoneum and the steep Trendelenburg position.

## References

[1] Novara G Ficarra V Rosen RC Systematic review and meta-analysis of perioperative outcomes and complications after robot-assisted radical prostatectomy. *Eur Urol* 2012; 62:431–452. 22749853 10.1016/j.eururo.2012.05.044

[2] Lestar M Gunnarsson L Lagerstrand L Hemodynamic perturbations during robot-assisted laparoscopic radical prostatectomy in 45 degrees Trendelenburg position. *Anesth Analg* 2011; 113:1069–1075. 21233502 10.1213/ANE.0b013e3182075d1f

[3] Meininger D Westphal K Bremerich DH Effects of posture and prolonged pneumoperitoneum on hemodynamic parameters during laparoscopy. *World J Surg* 2008; 32:1400–1405. 18224479 10.1007/s00268-007-9424-5

[4] Kalmar AF Dewaele F Foubert L Cerebral haemodynamic physiology during steep Trendelenburg position and CO(2) pneumoperitoneum. *Br J Anaesth* 2012; 108:478–484. 22258202 10.1093/bja/aer448

[5] Schramm P Treiber AH Berres M Time course of cerebrovascular autoregulation during extreme Trendelenburg position for robotic-assisted prostatic surgery. *Anaesthesia* 2014; 69:58–63. 24256501 10.1111/anae.12477

[6] Shikanov S Desai V Razmaria A Robotic radical prostatectomy for elderly patients: probability of achieving continence and potency 1 year after surgery. *J Urol* 2010; 183:1803–1807. 20299041 10.1016/j.juro.2010.01.016

[7] Collier A Ghosh S McGlynn B Prostate cancer, androgen deprivation therapy, obesity, the metabolic syndrome, type 2 diabetes, and cardiovascular disease: a review. *Am J Clin Oncol* 2012; 35:504–509. 21297430 10.1097/COC.0b013e318201a406

[8] Antonelli M Levy M Andrews PJ Hemodynamic monitoring in shock and implications for management. International Consensus Conference, Paris, France, 27–28 April 2006. *Intensive Care Med* 2007; 33:575–590. 17285286 10.1007/s00134-007-0531-4

[9] Monk TG Saini V Weldon BC Anesthetic management and one-year mortality after noncardiac surgery. *Anesth Analg* 2005; 100:4–10. 15616043 10.1213/01.ANE.0000147519.82841.5E

[10] Walsh M Devereaux PJ Garg AX Relationship between intraoperative mean arterial pressure and clinical outcomes after noncardiac surgery: toward an empirical definition of hypotension. *Anesthesiology* 2013; 119:507–515. 23835589 10.1097/ALN.0b013e3182a10e26

[11] Monge Garcia MI Gil Cano A Diaz Monrove JC Brachial artery peak velocity variation to predict fluid responsiveness in mechanically ventilated patients. *Crit Care* 2009; 13:R142. 19728876 10.1186/cc8027 PMC2784351

[12] Chin JH Lee EH Hwang GS Prediction of fluid responsiveness using dynamic preload indices in patients undergoing robot-assisted surgery with pneumoperitoneum in the Trendelenburg position. *Anaesth Intensive Care* 2013; 41:515–522. 23808512 10.1177/0310057X1304100413

[13] Monge Garcia MI Gil Cano A Gracia Romero M Dynamic arterial elastance to predict arterial pressure response to volume loading in preload-dependent patients. *Crit Care* 2011; 15:R15. 21226909 10.1186/cc9420 PMC3222048

[14] Monge Garcia M Gracia Romero M Gil Cano A Dynamic arterial elastance as a predictor of arterial pressure response to fluid administration: a validation study. *Crit Care* 2014; 18:626. 25407570 10.1186/s13054-014-0626-6 PMC4271484

[15] Swets JA Measuring the accuracy of diagnostic systems. *Science* 1988; 240:1285–1293. 3287615 10.1126/science.3287615

[16] Falabella A Moore-Jeffries E Sullivan MJ Cardiac function during steep Trendelenburg position and CO_2_ pneumoperitoneum for robotic-assisted prostatectomy: a trans-oesophageal Doppler probe study. *Int J Med Robot* 2007; 3:312–315. 18200624 10.1002/rcs.165

[17] Kim MS Bai SJ Lee JR Increase in intracranial pressure during carbon dioxide pneumoperitoneum with steep trendelenburg positioning proven by ultrasonographic measurement of optic nerve sheath diameter. *J Endourol* 2014; 28:801–806. 24517270 10.1089/end.2014.0019

[18] Hong JY Oh YJ Rha KH Pulmonary edema after da Vinci-assisted laparoscopic radical prostatectomy: a case report. *J Clin Anesth* 2010; 22:370–372. 20650386 10.1016/j.jclinane.2009.05.010

[19] Vos JJ Kalmar AF Struys MM Comparison of arterial pressure and plethysmographic waveform-based dynamic preload variables in assessing fluid responsiveness and dynamic arterial tone in patients undergoing major hepatic resection. *Br J Anaesth* 2013; 110:940–946. 23348202 10.1093/bja/aes508

[20] Pinsky MR Heart–lung interactions during positive-pressure ventilation. *New Horiz* 1994; 2:443–456. 7804794

[21] Phong SV Koh LK Anaesthesia for robotic-assisted radical prostatectomy: considerations for laparoscopy in the Trendelenburg position. *Anaesth Intensive Care* 2007; 35:281–285. 17444322 10.1177/0310057X0703500221

[22] Renner J Gruenewald M Quaden R Influence of increased intra-abdominal pressure on fluid responsiveness predicted by pulse pressure variation and stroke volume variation in a porcine model. *Crit Care Med* 2009; 37:650–658. 19114894 10.1097/CCM.0b013e3181959864

[23] Mesquida J Kim HK Pinsky MR Effect of tidal volume, intrathoracic pressure, and cardiac contractility on variations in pulse pressure, stroke volume, and intrathoracic blood volume. *Intensive Care Med* 2011; 37:1672–1679. 21739340 10.1007/s00134-011-2304-3 PMC3818902

[24] Bouchacourt JP Riva JA Grignola JC The increase of vasomotor tone avoids the ability of the dynamic preload indicators to estimate fluid responsiveness. *BMC Anesthesiol* 2013; 13:41. 24215252 10.1186/1471-2253-13-41 PMC4175099

[25] Perner A Faber T Stroke volume variation does not predict fluid responsiveness in patients with septic shock on pressure support ventilation. *Acta Anaesthesiol Scand* 2006; 50:1068–1073. 16939480 10.1111/j.1399-6576.2006.01120.x

[26] Rosendal C Markin S Hien MD Cardiac and hemodynamic consequences during capnoperitoneum and steep Trendelenburg positioning: lessons learned from robot-assisted laparoscopic prostatectomy. *J Clin Anesth* 2014; 26:383–389. 25086483 10.1016/j.jclinane.2014.01.014

[27] Cecconi M Monge Garcia MI Gracia Romero M The use of pulse pressure variation and stroke volume variation in spontaneously breathing patients to assess dynamic arterial elastance and to predict arterial pressure response to fluid administration. *Anesth Analg* 2015; 120:76–84. 25230102 10.1213/ANE.0000000000000442

[28] Kelly RP Ting CT Yang TM Effective arterial elastance as index of arterial vascular load in humans. *Circulation* 1992; 86:513–521. 1638719 10.1161/01.cir.86.2.513

[29] Guinot PG Bernard E Levrard M Dynamic arterial elastance predicts mean arterial pressure decrease associated with decreasing norepinephrine dosage in septic shock. *Critical care* 2015; 19:14. 25598221 10.1186/s13054-014-0732-5 PMC4335631

[30] Hadian M Pinsky MR Evidence-based review of the use of the pulmonary artery catheter: impact data and complications. *Crit Care* 2006; 10 Suppl 3:S8. 17164020 10.1186/cc4834 PMC3226129

[31] Chan KL Cohen GI Sochowski RA Complications of transesophageal echocardiography in ambulatory adult patients: analysis of 1500 consecutive examinations. *J Am Soc Echocardiogr* 1991; 4:577–582. 1760179 10.1016/s0894-7317(14)80216-2

[32] Seward JB Khandheria BK Oh JK Critical appraisal of transesophageal echocardiography: limitations, pitfalls, and complications. *J Am Soc Echocardiogr* 1992; 5:288–305. 1622623 10.1016/s0894-7317(14)80352-0

